# An Integrated Pulsation-Free, Backflow-Free Micropump Using the Analog Waveform-Driven Braille Actuator

**DOI:** 10.3390/mi13020294

**Published:** 2022-02-13

**Authors:** Kotaro Nishikata, Masataka Nakamura, Yuto Arai, Nobuyuki Futai

**Affiliations:** Department of Mechanical Engineering, Shibaura Institute of Technology, 3-7-5 Toyosu, Koto-ku, Tokyo 135-8548, Japan; md20071@shibaura-it.ac.jp (K.N.); md21082@shibaura-it.ac.jp (M.N.); aa16002@shibaura-it.ac.jp (Y.A.)

**Keywords:** Braille microfluidics, integrated micropump, piezoelectric pump, constant flow waveform, perfusion culture, elastomeric pump

## Abstract

The widespread adoption of long-term organs-on-a-chip culture necessitates both active perfusions that mimic physiological flow conditions and minimization of the complexity of microfluidic system and fluid handling. In particular, flow in microtissue such as microvascular is free of pulsation and backflow. The refreshable Braille actuator-based integrated microfluidic system can be employed with simple microchannels and setups. However, due to high pulsatile flow and backflow, ordinary Braille-driven micropumps generate non-physiological flow conditions. We have described a simple method for creating steady flow employing Braille actuators driven with a high-voltage analog waveform, called “constant flow waveform”, without incorporating complicated structures into the microchannel or actuator. We determined the constant flow waveform by measuring volume change of microchannel caused by actuated Braille pins using a conventional fluorescent dye and microscope. Using the constant flow waveform, we demonstrated that a Braille-driven pump reduced pulsating flow by 79% and backflow by 63% compared to conventional Braille-driven pump. Furthermore, we demonstrated that a parallel pair of three-stranded pin pumps effectively eliminated backflow by driving two pumps with the constant flow waveform half-cycle shifted to each other. Moreover, by raising the driving frequency, we could increase the average flow rate to ~2× higher than previously reported flow rate of a typical Braille-driven micropump.

## 1. Introduction

Mimicking the complex functions of living organs in a small space, frequently called organs-on-a-chip [1,2], is crucial in life science research. These miniaturized devices serve as platforms for high-throughput disease modeling [3,4] and drug screening [5,6]. Circulation is required to maintain the metabolic status of organs and cells in vivo. Therefore, an active micropump is an essential component to constitute an organs-on-a-chip with circulation. Although several passive microfluidic pumps have been successfully demonstrated [7], it is difficult to reproduce the circulation. Organs-on-a-chip associated with recirculating perfusion mimics physiological conditions more closely [8]. Thus, advanced organ-on-chip systems should stimulate circulation through active micropumps and valves to regulate the flow within the chip.

Microflow in perfusion cell culture is significant for mimicking physiological flow conditions and facilitating the construction of organs-on-a-chip. For instance, wall shear stress (WSS) on endothelial cells significantly affects cell shape and lumen formation [9,10,11]. Additionally, the production of self-assembled organ-like cell culture in the extracellular matrix (ECM) often necessitates a long-term culture with perfusion [12,13]. The use of microfluidic chips combined with several external pumps has been effectively applied to various cellular assays [14]. Examples include the perfusion of vascularized cancer spheroids in a microfluidic system attached to a syringe pump [15] and a piezoelectric or pneumatic pumping system that generates pulsatile flow for evaluating endothelial cell response [16,17]. The pulsation of the peristaltic pump may be adequate for organ-on-a-chips for arterial endothelial cells. In contrast, it is not suitable for capillary networks, where the interstitial flow dominates.

Minimizing the complexity of the fluid handling is critical in a contamination-free long-term microfluidic culture with real-time live imaging. Overall, micropumps integrated on a chip offer advantages over passive chips attached to syringe pumps or other non-microfluidic pump components. For example, fluid control through microchannel displacement using a pneumatic channel is referred to as large-scale microfluidic integration and is widely employed [18]. Moreover, pneumatic micropumps with check valves and fluid capacitors integrated into the microchannel are used [19,20]. Other actuators have been effectively employed as integrated micropumps, such as electromagnetic actuation of elastomeric microchannels with nozzles and diffusers [21,22], pumping liquid by driving magnetic beads encapsulated in a microfluidic channel [23], peristaltic action of three piezoelectric benders [24], stirrer-based kinetic pump integrated microfluidic plate [25], and peristaltic pumps integrated into a thermoplastic elastomer disc [26]. A comparison of the reported micropumps intended for microfluidic perfusion cell culture is listed in Table 1.

Among various types of microvalves and micropumps implemented as deformation of the elastomeric microchannel, the integrated microfluidics using commercially available refreshable Braille displays offers additional advantages in having a good balance of actuation force and density in valve/pump placement, a simple control technique, and low-cost availability [27]. Applications include perfusion culture [27,28,29,30], flow cytometry [31], embryo culture [32], plug flow formation for drug screening [33], micromixers [34], and particle sorting [35]. However, a Braille-driven micropump generates significant pulsatile flows due to only two (i.e., up and down) positions of Braille pins. Previous studies that employed a Braille-driven microfluidic system either took advantage of pulsatile flow for mixing, used a diffuser to stabilize flow, or just as valves that switched flows from other external pumps. Diffusers and microsized check valves have a limited effect on backflow and pulsatile flow suppression, making priming difficult. Therefore, all previously developed integrated micropumps based on commercially available actuators generate pulsatile flow or backflow, increase structural complexity that affects long-term perfusion culture, or both. The inability to regulate the volume change of the elastomeric microfluidic channel is the fundamental cause of flow pulsation by peristaltic pumps, including Braille-driven micropumps.

We present a Braille-driven micropump that generates steady flows without any structural alteration. In our approach, we drove Braille actuators with waveforms making the Braille pin tips to exclude liquid in the elastomeric microchannel with straight or circular seat shapes at a constant rate. In this paper, we refer to the waveform as a constant flow waveform, from now on abbreviated as CFW. The CFW is obtained by measuring fluorescence inside a microchannel deformed by a Braille pin driven with ramp voltage. Driving three pins of a micropump with CFWs significantly reduced pulsation. Backflow was eliminated using a parallel pair of CFW-driven three-pin pumps (i.e., six-pin pump). Furthermore, we showed a non-alternating flow with six Braille pins driven with four CFWs.

## 2. Material and Methods

### 2.1. Braille-Driven Flow in Microchannels

**Figure 1:**
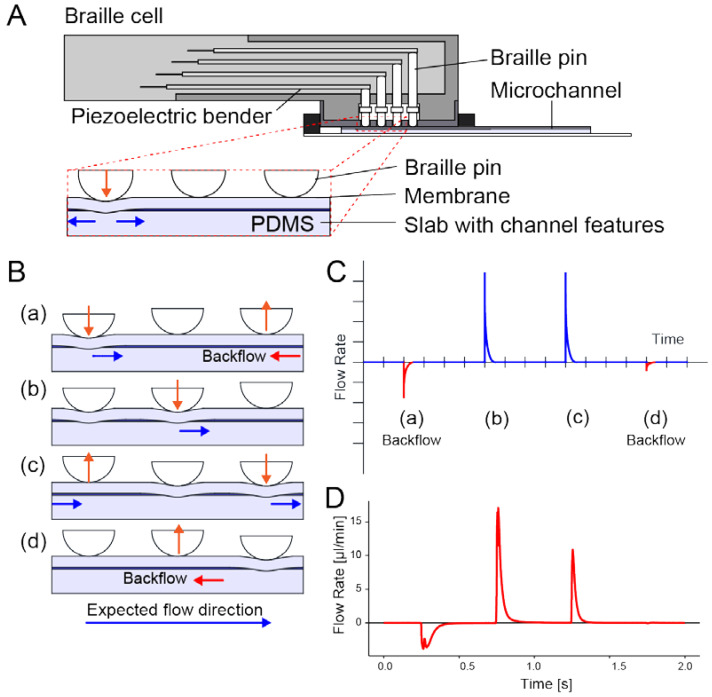
An integrated micropump driven by pins of a refreshable Braille display. (**A**) Installation of a Braille pin micropump. By applying a voltage to the corresponding piezoelectric bender, a Braille pin generates a downward force. The downward force temporarily occludes the elastomeric microchannel, displacing the liquid inside. (**B**) A three-stranded Braille pin micropump scheme. Peristaltic actions repeating steps (**a**–**d**) feed the liquid from left to right. Orange arrows indicate pin movement; blue arrows indicate the direction of transient flow generated by the pin movement. (**C**) Illustration of an instantaneous flow rate of a three-stranded Braille pin micropump driven with the scheme shown in (**B**). Symbols (**a**–**d**) corresponding to those in (**B**). (**D**) In a microfluidic device employed, we observed a typical instantaneous flow rate of a 3-pin Braille pin pump (See Supplementary Figure S2). The refresh rate of the steps was two steps/s.

The microfluidic pump comprises a microfluidic chip and an actuator array called a “Braille cell”. The microfluidic chip is made of poly(dimethylsiloxane) (PDMS) and fabricated by a modified soft lithographic method that produces microchannels with bell-shaped sidewalls [36]. The Braille cell is a 2 × 4 matrix of pins actuated by piezoelectric benders that express one Braille character. In this study we used a type of Braille cells that has voltage inputs directly connected to the piezo bender electrodes (SC9, KGS, Saitama, Japan). A Braille cell’s fingerboard fits into a fixture bonded to the substrate. As depicted in Figure 1A, the Braille pins can displace the microchannel beneath and generate flows therein.

To pump liquid in a microchannel, three or more Braille are driven to execute the peristaltic action on the microchannel depicted in Figure 1B. Because a Braille pin can be fully protruded or retracted, the peristaltic motions of Braille pins on a microchannel cause significant pulsation and backflow, as illustrated in Figure 1C,D that depicts a typical waveform of instantaneous flow rate downstream of a conventional Braille drive scheme shown in Figure 1B, measured using a method described in Section 2.5.

In general use, since a high-voltage switch array in a Braille cell backplane only provides “on-or-off” drive, either 0 or 200 V is applied to piezoelectric benders to displace Braille pins. In this study, we did not employ a backplane but instead used high-voltage amplifiers to apply an arbitrary voltage to the piezoelectric bimorph actuators of a Braille cell. The displacement of the bimorph actuator was controlled by importing a voltage waveform from the Analog Output Generator in MATLAB (MathWorks, Natick, MA, USA) and amplifying it with the data acquisition device (cDAQ-9191 and NI9263, National Instruments, Austin, TX, USA) and piezoelectric amplifiers (SVR 500/3 and SVR 350/3, Piezosystem Jena, Jena, Germany).

As shown in Supplementary Figure S2, a microfluidic device illustrated in Figure 1A comprises a PDMS channel feature, a PDMS membrane, a fingerplate fixture, and a glass substrate. The region for cell culture is at the stem of the Y-shaped channel downstream of the parallel pair of two 3-pin pumps. The outlet of the perfusion (i.e., downstream of the 6-pin pump) is also used as the inlet of cell suspensions to seed cells into the channel. Typical soft lithographic techniques were employed to fabricate PDMS channel features. A film photomask for lithography was designed using AutoCAD 2019 or recent versions (Autodesk, San Rafael, CA, USA). The channel was drawn as 300 µm-wide lines, with 800 µm-diameter filled circles overlaid at the middle pin position of circular seats. The PDMS channel features were fabricated as previously described [37]. Briefly, PDMS (KE-106, Shin-Etsu Chemical, Tokyo, Japan) was cast against a channel mold fabricated on glass by patterning SU-8 3050 photoresist (Nippon Kayaku, Tokyo, Japan). A 300-µm-thick PDMS membrane was created with spin coating. The cast PDMS was cured at 65 °C for 3 h, followed by at 120 °C for 10 min. A 1.5 mm diameter inlet, and outlet holes were formed on the membrane using a biopsy punch. Both PDMS layers and a 450 µm-thick glass substrate (No.5 coverglass, Matsunami, Osaka, Japan) bonded after vacuum air plasma treatment. Finally, a poly(lactic acid) (PLA) Braille fingerplate fixture was 3D printed (Ultimaker 3, Ultimaker, Utrecht, The Netherlands) and attached to the substrate with epoxy glue and manual alignment.

### 2.2. Measurement of Exclusion Volume

We measured the change in channel volume by driving the Braille pin to determine the voltage waveforms applied to Braille actuators. A 30-μM solution of sodium fluorescein (Kanto Chemical, Tokyo, Japan) was introduced into the microfluidic channel. The microchannel directly beneath the Braille pin was photographed using a fluorescence microscope (DMi8, Leica, Wetzlar, Germany) and CMOS camera (DMK33UX174, The Imaging Source, Bremen, Germany), and the captured video was sliced using ImageJ. The intensity in the sliced images was converted to the thickness of the fluorescent dye solution in the channel. The thickness values were then integrated over the area of the channel to obtain the volume of the channel. The MATLAB script used to calculate the excluded volume is shown in Supporting Information (SI.3). The fluorescence intensity follows Beer’s law, which states that the absorbance of the transmitted light by fluorophores at a constant concentration is proportional to the thickness of the liquid (i.e., the channel height). The Braille pins used for imaging were painted black so that the change in fluorescence intensity caused by the movement of the Braille pins was minimized. Supplementary Video S1 shows typical images of a microchannel deformed by a Braille pin. We applied a simple triangular wave voltage waveform to the bimorph actuator.

Then, we plotted a graph of the excluded volume against the applied voltage. The approximate equation yielded a constant flow waveform (CFW). The CFW is a voltage waveform applied to Braille actuators resulting in a continuous change in microfluid volume.

### 2.3. Determination of CFWs Using Fluid-Structure Interaction Simulation

We constructed a nonlinear structural model of a PDMS microchannel and a pin tip that deforms the microchannel to investigate the feasibility of CFWs using numerical simulations. Then, we performed a fluid-structure interaction (FSI) analysis of a Braille micropump.

First, the geometry of a 300 μm-wide, 50 μm-thick microchannel with a bell-shaped cross-section [36] was modeled using Autodesk Inventor 2018 or recent versions (Autodesk) and imported into ANSYS 2020 R2 (ANSYS, Canonsburg, PA, USA) for FSI analysis. We employed previously reported Sylgard 184 material properties [38] to model the elastomer zone that produces a microchannel. Typical simulation settings and 3D models are shown in Supporting Information (SI.4).

Next, a Braille pin tip was modeled as a rigid body and displaced toward the channel. We then evaluated the volume of the fluid zone inside the channel as well as the flow generated due to deformation. Since we did not model a piezoelectric actuator in ANSYS, the displacement of the Braille pin tip was converted to the voltage that would be applied to the Braille pin using the quadratic function obtained from the following measurement: A digital camera (Nikon 1 J4 with a telecentric lens, Nikon, Tokyo, Japan) was used to record the position of the bottom end of the Braille pin with applied voltage ranging from 40–200 V in 10 V increments. The displacement of the Braille pin was measured from the images using AutoCAD. Data points relating the actuation voltage to the Braille pin displacement were fitted with a quadratic function.

**Figure 2:**
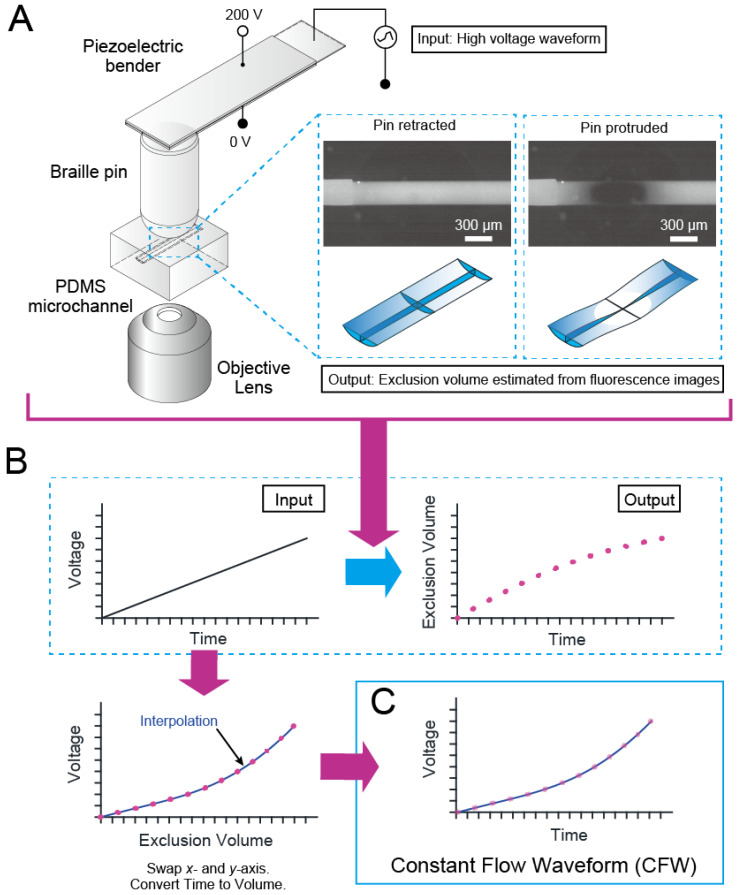
Generation of the constant flow waveform (CFW) for a pair of a single Braille pin and an elastomeric bell-shaped microchannel. (**A**) Setup for measurement of exclusion volume. The microchannel under test is filled with a fluorophore solution and placed on a stage of an inverted microscope with a Braille pin on its top surface. The fluorescence decreases as the voltage applied to the Braille pin increases. From Beer’s law, we can obtain values proportional to the exclusion volume as an intensity change. (**B**) Steps for determination of a CFW. First, we have the voltage input and the exclusion volume output as a function of time. We then plot the voltage along the y-axis and the exclusion volume along the linear x-axis. Associating the time to the linear scale of exclusion volume gives the time-varying voltage that makes a constant change of exclusion volume, i.e., flow rate. (**C**) Typical CFWs for single Braille pin strokes (i.e., one pin protrusion followed by a retraction) on microchannels with different widths.

Finally, we obtained a CFW by interpolating the relationship between the voltage and the fluid zone volume decrease (i.e., exclusion volume). See Figure 2B.

### 2.4. Pump Scheme Determination

By activating the downstream pin before retracting the middle pin, we can avoid a significant backflow of a three-stranded pin pump. However, backflow still occurs when the downstream pin is retracted. To offset the backflow with a more significant forward flow, we should activate the middle pin while retracting the downstream pin. To ensure the scheme above, we need to ensure successful occlusion by pin activation and reduce the backflow volume generated by downstream pin retraction. Therefore, the channel widths on the downstream and upstream sides were narrowed down to 300 µm. However, the exclusion volume in a straight 300 µm-wide channel is not large enough to suppress the backflow caused by the retraction of the downstream pin. Therefore, we placed a circular seat on the middle pin tip to displace a larger volume than that generated from the straight channel.

Furthermore, to reduce flow pulsation, the flows generated by both pins should cancel out at a constant rate, as shown in Supplementary Figure S1A, thus these pins should be driven by anti-phase CFWs without blanking, as shown in Supplementary Figure S1B. However, the middle pin should displace a significant volume to increase the total flow rate, and thus retraction of the middle pin should be rapid, such that it is fully retracted while the downstream pin is closed and upstream pin is opened, as shown in Supplementary Figure S1C. Therefore, the state with the upstream closed and the downstream open should be at the mid-period of the CFW of the middle pin. As shown in Supplementary Figure S1D, the middle pin should be retracted quickly so that it occurs while the upstream pin is open and downstream is closed.

A three-stranded pin pump-driven with CFWs can reduce pulsation and backflow but cannot eliminate it. Therefore, we propose a parallel pair of two three-stranded pin pumps. The same waveform drives the two pumps with half-cycle shifted to each other.

### 2.5. Measurement of Flow Rates

We measured the flow rate of Braille-driven micropump to assess the effect of waveform selection on flow pulsation or backflow. We develop a Braille cell, a microfluidic device, and a controller described in Section 2.2.

Polystyrene 2.0 μm fluorescent microbeads (FSDG005, Bangs Laboratory, Fishers, IN, USA) were inserted into the microchannel shown in Supplementary Figure S2. We captured fluorescence images inside the channel during pumping using IC Capture (The Imaging Source) at 400 frames per second. Supplementary Video S2 shows typical images of microspheres flowing in a microchannel. The images were binarized using ImageJ software. Flow velocity in the microchannel was obtained from the binarized images with PIVlab [39], a particle image velocimetry tool for MATLAB.

The temporal flow velocity data v[n] (0≤n≤N for one period) was converted to a time series of instantaneous flow rate Q[n] by multiplying the cross-sectional area of the channel A:

Q[n]=Av[n].

The backflow ratio $R_{back}$ in the period T was calculated as the volume of backflow divided by the total absolute value of the volume flowing into the channel. To evaluate pulsation of flow, we calculated the coefficient of variation (CV) of flow rate from temporal evolution data of flow rate:

(1) $$R_{back}=\frac{\sum_{n=0}^{N}H\left(-Q\left[n\right]\right)Q\left[n\right]}{\bar{Q}}$$

where H(x) is a unit step function and $\bar{Q}$ the mean flow rate in one period:

(2) $$\bar{Q}=\frac{1}{N}\overset{N}{\underset{n=0}{\sum }}\left|Q\left[n\right]\right|$$

The CV of flow rate was given by

(3) $$CV=\frac{\sigma_{Q}}{\bar{Q}}$$

where $\sigma_{Q}$ is the standard deviation of absolute values

(4) $$\sigma_{Q}=\sqrt{\frac{1}{N}\overset{N}{\underset{n=0}{\sum }}{\left(\left|Q\left[n\right]\right|-\bar{Q}\right)}^{2}}$$

## 3. Results and Discussions

### 3.1. Generation of the CFW

We developed a method for obtaining a voltage waveform applied to a Braille actuator that causes the displacement of a single Braille pin to generate flow inside a channel at a constant flow rate, called “CFW”.

Figure 2 depicts the principle and steps for determining a CFW experimentally. Overall, a Braille pin generates flow in an elastomeric microfluidic channel when the pin exerts a force to alter the channel’s volume, or it unloads to make the channel regain its original shape. The volume change rate caused by the pin movement must be consistent to make the flow rate constant.

As shown in Figure 2A, we developed a system to measure the channel volume change due to pin movement. The system comprises a Braille actuator connected with piezo amplifiers, an elastomeric microchannel containing fluorescent dye solution, and an inverted fluorescence microscope. Figure 2B depicts how a CFW is obtained. First, we measured the fluorescence intensity of the channel with voltage waveform input to the piezo amp using the system shown in Figure 2A. We established the relationship between the input voltage and the exclusion volume with time as the parameter because the decrement of the fluorescence intensity can be considered proportional to the volume excluded from the channel due to the Braille pin. The CFW, which produces a constant rate of exclusion volume over time, can be determined as a regression function of the relationship between the exclusion volume as the dependent variable and the voltage as the independent variable.

**Figure 3:**
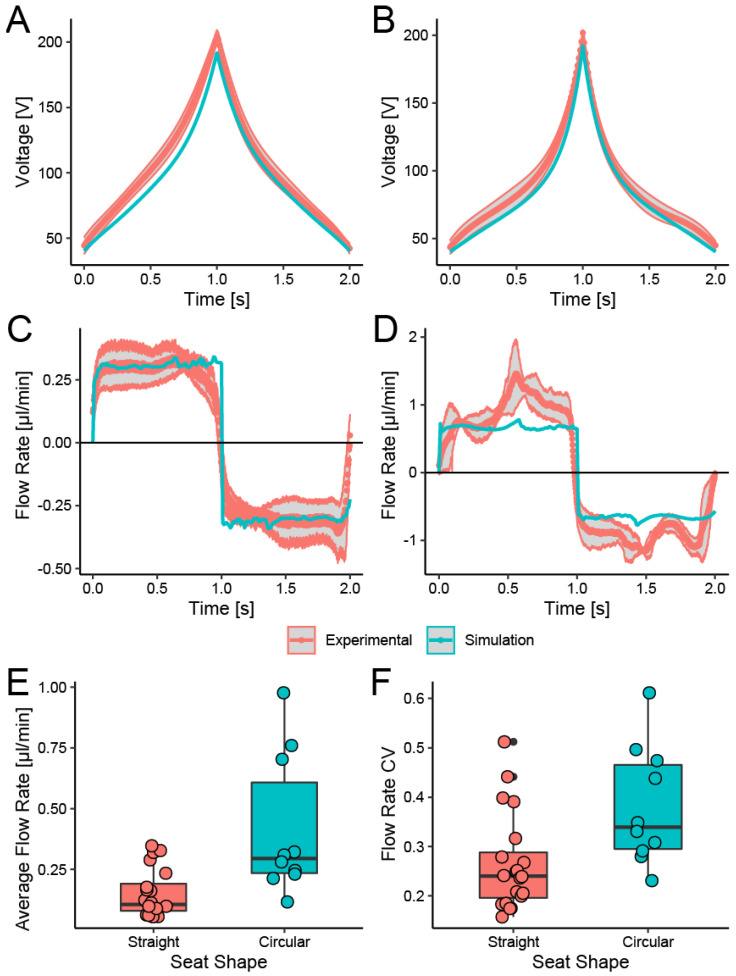
Constant flow waveforms (CFWs) and their flow rate output. The CFWs are for elastomeric 50 µm-height bell-shaped microfluidic channel with a Braille pin. The CFWs obtained from the simulation result were shown in dashed lines; CFWs from the exclusion volume measurement were shown in solid lines. (**A**,**B**) average and 95% confidence band of measured CFWs, and a CFW obtained from simulation. The CFWs obtained from the simulation result were shown in green dashed lines; CFWs from the rejection volume measurement were shown in red solid lines. (**C**,**D**) Instantaneous flow rate generated by Braille pin drive using CFWs and 95% confidence band of measured flow rate. (**A**,**C**) are of straight channels; (**B**,**D**) wide channels with a φ800 μm circular seat. (**E**) Average flow rate of one cycle of CFW-driven flow shown in (**C**,**D**). (**F**) The coefficient of variation (CV) of the flow rate fluctuation.

Typical CFWs for two different PDMS microchannels are shown in Figure 3A,B. The waveform slope is slight at the initial stage, and it increases over time as the pin displacement increases. The slight initial slope indicates the small mechanical compliance of the channel membrane (i.e., it gives a large displacement with a small force exerting on it) and a force-displacement relationship of a piezo bimorph actuator (i.e., it can generate a large force when the displacement is small). However, when the cross-sectional size of a microchannel is so tiny that it is occluded fully by the Braille pin, the slope of the CFW then decreases again slightly before it reaches the maximum. Therefore, the CFW cannot be expressed as a simple straight line or curve, but it has an inflection point. We found a 6th-order polynomial regression was required to estimate CFWs from experimental results.

Figure 3A,B also shows that CFWs obtained through the experiment described in Section 2.2 are in reasonable agreement with that obtained through the simulation described in Section 2.3. It suggests that fluid-structure analysis can generate CFWs before device fabrication or when there is no setup fluorescence imaging.

### 3.2. Pulsation Reduction with CFWs

We evaluated the stability of the flow generated by an up-down stroke of one Braille pin driven with CFWs obtained by experiment and simulation. We also examined a channel with a circular seat over the middle pin with a larger inner volume than that of the straight channel.

Figure 3C,D shows the instantaneous flow rate through a straight and circular seat PDMS channel during a Braille pin displaces each channel with an up-down stroke according to a CFW shown in Figure 3A. For both experimental results and FSI simulation results, a stable square wave-like flow with low fluctuation was obtained. A comparison of the average flow rate generated from a straight and circular seat channel is shown in Figure 3E. A circular seat channel generates approximately 3× higher average flow rate with more considerable variation than a straight channel. A large flow rate with a significant variation of a circular seat channel can be explained by its larger channel capacity that displaces a larger but less stable volume of liquid inside. The CV of the flow rate was lower than 0.5 for most cases, as shown in Figure 3F.

### 3.3. Flow Generated by Single and Parallel Braille Pin Pumps

We measured the flow generated by a micropump consisting of three Braille pins driven with CFWs (Hereinafter “three-pin pump”) and a parallel pair of two three-pin pumps (“six-pin pump”). We obtained the CFWs used to drive the Braille pins by the experiment described in Section 2.2, and three or six pins were driven following the scheme described in Section 2.4.

**Figure 4:**
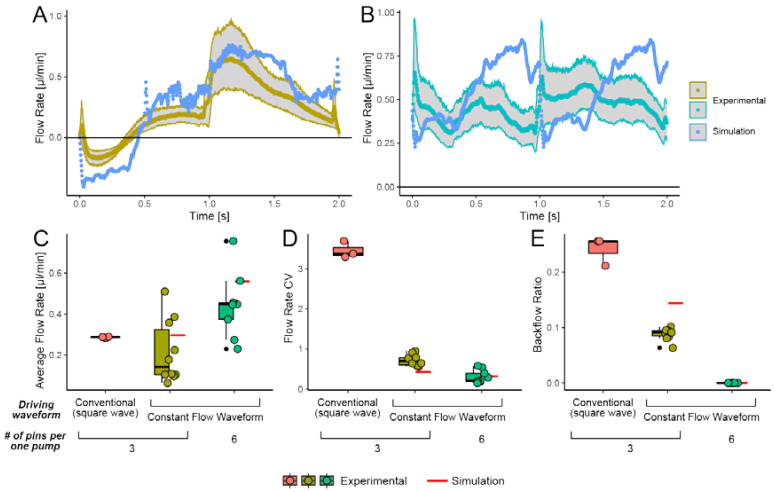
Comparison of flows generated by three-stranded Braille pins driven with CFWs (“three-pin pump”) and a parallel pair of two three-pin pumps (“six-pin pump”). (**A**,**B**) Instantaneous flow rate of one set of three-pin pumps (**A**) and six-pin pumps (**B**) within one period (2 s). Mean values and 95% confidence intervals for multiple device samples (N=3 for square wave-driven three-pin pumps; N=11 for CFW-driven three-pin pumps; N=10 for six-pin pumps) are shown. (**C**) Average flow rate, (**D**) coefficient of variance (CV), and (**E**) Backflow ratio of three- and six-pin pumps.

Figure 4A depicts the instantaneous flow rate observed downstream of three-pin pumps. Although the instantaneous flow rate in one cycle still has slight pulsation at the end of the cycle (*t* ~ 2.0 s), the pulsation is reduced by 76% compared to conventional Braille drive shown in Figure 1D. Additionally, the backflow ratio is reduced by 63% compared to the conventional Braille drive, as shown in Figure 4E. The measured flow rate and that obtained from the simulation display similar tendencies. However, the amplitude of the measured value was smaller in both the forward and reverse directions. The differences between simulation and experimental results are probably due to the non-ideal channel occlusion. Figure 4B depicts the instantaneous flow rate generated by a six-pin pump. Since the six-pin pump comprises two three-stranded Braille pin pumps in parallel and operated, out-of-phase (i.e., the waveforms of the three pins of one pump are shifted by one-half-cycle relative to that of the three pins of another pump), the period of the flow rate is half that of the drive waveform. The variations between the flow rates obtained by simulation and experiment shown in Figure 4A can explain that observed in Figure 4B by overlaying two waveforms half a cycle off each other.

Figure 4C depicts that the mean flow rate of a six-pin pump was approximately doubled compared to that of three-pin pumps driven by either conventional square wave drive or CFW drive. As expected, Figure 4D shows that the CFW-driven Braille pumps had significantly low CV values than square wave-driven pumps; CV values of six-pin CFW-driven pumps were less than half, compared to that of a three-pin pump. Additionally, we could eliminate backflow with six-pin pumps as expected. As shown in Figure 4E, the backflow ratio, $R_{back}$, was zero for six-pin pumps. The reduction of the backflow ratio can be explained by the gradual valving that enabled canceling out of significant backflow produced by the upstream with the forward flow by other pins. Most backflow originates from the downstream pin regardless of the waveform applied to Braille actuators. In the case of CFWs, backflow was minimized due to some overlaps in the waveforms driving the downstream and middle pins.

### 3.4. Flowrates with Increased Pin Refresh Rates

Overall, increasing the pumping frequency can increase the flow rate. We measured the flow rate of CFWs that drive parallel (six-pin) Braille pin pumps at varying frequencies.

**Figure 5:**
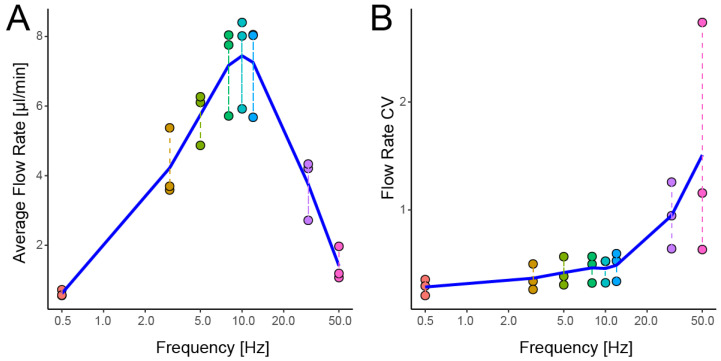
Dependence of flowrates of a parallel Braille pin pump pair on the driving frequency. (**A**) Average flow rate, and (**B**) the coefficient of variation in temporal variations of the instantaneous flow rate of a 6-pin pump in the range of 0.5~50 Hz driving frequencies. A blue segmented line in each panel denotes the mean value of multiple points grouped by the frequency.

Figure 5 depicts the frequency dependence of CFW-driven flow ranging from 0.5–50 Hz. As shown in Figure 5A, the average flow rate increased with frequency, reaching a maximum value of 7.4 ± 1.3 µL/min (mean ± SD) at 10.0 Hz. The flow rate was approximately two times higher than the previously reported maximum flow rate with a Braille pin pump of 2.94 µL/min [28]. The flow rate gradually decreased as the driving frequency increased, reaching 1.4 ± 0.5 µL/min at 50 Hz. It implies that the drive frequency is regarded as the upper limit of 10 Hz. Figure 5B depicts that the flow rate’s CV increased with frequency. The most likely cause of elevated flow rate CV at a high frequency is thought to be that the channel deformation (i.e., switching of open or closed status) response to high-frequency excitation by Braille pins is delayed, and therefore backflow increases.

However, the upper limit of the driving frequency rather depends on the performance of high-voltage amplifiers that drive piezoelectric actuators following CFWs. Overall, it is extremely difficult to downsize and reduce the power consumption of a high-voltage amplifier without narrowing the frequency band. According to our expertise and knowledge of electronics, a practical upper limit of the driving frequency of a compact (in a printed circuit board of 1–10 cm^2^) 200 *V* analog amplifier should be approximately 10 Hz. In this case, the maximum flow rate we can propose is 7.4 µL/min, which is adequate for most on-chip cell culture applications. For instance, vascularized spheroids were perfused at 0.5 µL/min for four days [15]. However, a flow rate of more than 7.4 µL/min will be required for studies focusing on the impact of flow rate or WSS on cell cultures, such as comparing several gene expressions of spheroids cultured under 1 µL/min and 10 µL/min [40]. Further studies on determining the optimum CFW at different frequencies are necessary. The CFW-based Braille micropump drive method proposed in this study can overcome the problems of microvascular culture using a passive pump [9,12,13], such as the inability to circulate the culture medium and the decline in flow rate with time, thus enabling more physiological and long-term perfusion culture.

## 4. Conclusions

We proposed an integrated microfluidic pump using Braille actuators driven with CFWs. The CFW is a time series of the voltage applied to a Braille actuator, with the exclusion volume due to the pin movement changing at a constant rate. We obtained CFWs by measuring the change in fluorescence inside a microchannel while applying a variable voltage to a Braille pin. The CFWs obtained from the experiments are in close agreement with the CFWs obtained from the simulations, suggesting that CFWs can be generated by FSI analysis only, even when fluorescence imaging is not available. The flow rate at the channel exhibited a square wave-like temporal evolution when a pin protrudes and retracts in a CFW cycle.

Then, we analyzed a three-stranded pin pump-driven by different CFWs. As a result, the pulsatile flow was reduced by 76%, and the backflow was decreased by 63% compared to the conventional Braille drive. The six-pin configuration eliminated the backflow that the three-pin pump could not remove. As the driving frequency increased up to 10 Hz, the average flow rate increased. The average flow rate at 10 Hz was about twice as high as the average flow rate of a typical Braille-driven micropump previously reported.

Braille pin micropumps regulated with CFWs allow for the reproduction of physiological flow in Braille-based on-chip perfusion culture. In a compact and easy-to-use system, the CFW-based Braille actuation can produce a wide range of flow rates. It could be employed in several low-cost, mobile, and reliable cells- or organ-on-chip systems, such as long-term perfusion cultures system that do not require pulsation.

## Figures and Tables

**Table 1:** Comparison of micropumps intended for microfluidic perfusion cell culture.

Actuation Method	Structure for Backflow Prevention	Flow Rate [µL/min]	Driving Frequency Range [Hz]	Reference
Pneumatic	Check valve	87.6 ± 5.0	0.5~4.5	[19]
Pneumatic	Check valve	21.61	0.5~4.0	[20]
Magnet + Solenoid	Nozzle/diffuser	7	10~160	[21]
Magnet + DC motor	Nozzle/diffuser	342.4	1000~1600	[22]
Magnetic Beads	None	1.125 × F ± 0.011	F = 0~25	[23]
PZT bender	None	6	0~100	[24]
Magnetic Stirrer	None	-	~6500 [rpm]	[25]
Magnet + Stepper motor	None	43.3	~800 [rph]	[26]
Braille actuator	None	2.94	0.25~2.0	[28]
Braille actuator	Nozzle/diffuser	1.2	1	[31]
Braille actuator	None	7.4	0.5~50	This work

## Supplementary Materials

The following supporting information can be downloaded at: https://www.mdpi.com/article/10.3390/mi13020294/s1, Figure S1: Construction of waveforms for a three-stranded Braille pin pump. Each pin’s waveform is a repetition of the “constant flow rate” waveforms (CFWs) depicted in Figure 2. The waveform for the pin at the middle contains a CFW in the upstroke direction only, whereas the upstream and downstream pins were driven with both upstroke and downstroke CFWs. The waveforms for the upstream and downstream pin are shifted half-cycle to each other.; Figure S2: A Braille pump-driven microfluidic device used for flowrate evaluation. (A) An upper view of the microfluidic device without a Braille actuator. (B) Illustration of an exploded view of the microfluidic device with a Braille actuator attached; Figure S3: 3D model used for FSI analysis. (A,B) Models used for the analysis of the 1-pin pump. (C) Model used for the three-pin pump. (D) Model used for the six-pin pump; Figure S4: A parallel pair of two three-stranded Braille pin pump to eliminate backflow at the downstream. Two pins illustrated with waveforms of the same color denote that these pins were driven with an identical waveform; Table S1: Analysis Settings for Transient Structural Analysis; Table S2: Analysis Settings for Fluent; Table S3: Analysis Settings for System coupling; Video S1: Fluorescence images inside a microchannel using a Braille pin driven with triangular wave voltage waveform; Video S2: Fluorescence microbeads images inside the microchannel during pumping.
