# Supplementary material for: An Integrated Pulsation-Free, Backflow-Free Micropump Using the Analog Waveform-Driven Braille Actuator
Source: Micromachines (Basel). 2022 Feb 13;13(2):294. doi: 10.3390/mi13020294 (PMC8879040; doi:10.3390/mi13020294)
Supplement: Supplementary file 1 [file micromachines-13-00294-s001.zip › mm-Anaten-SI_round1.pdf]

micromachines

Supporting Information

An Integrated Pulsation-Free, Backflow-Free Micropump Using the Analog Waveform-Driven Braille Actuator

Kotaro Nishikata, Masataka Nakamura, Yuto Arai and Nobuyuki Futai

Department of Mechanical Engineering, Shibaura Institute of Technology, 3-7-5 Toyosu, Koto-ku, Tokyo 135-8548, Japan

SI. 1. Use of CFWs for a three-stranded Braille pin pump

When applying CFWs to a three-pin Braille pin pump, we assigned the function of liquid feeding to the pin at the middle and the function of the valve to the pins at the upstream and downstream. As described in Section 2.4, the configuration ensures a significant flow rate and backflow suppression.

Figure S1A depicts the preferred driving waveform for the pin at the downstream ("downstream pin") and the pin at the upstream ("upstream pin"). To reduce pulsation, we employed CFWs for both protruding and retracting directions with similar expected flow rates (i.e., the waveform is symmetrical to the origin of the time: t=0). The downstream and upstream pins must be alternating to establish pumping. Therefore, the waveforms for the downstream or upstream pins are shifted half-cycle to each other.

However, a CFW is employed to drive the pin at the middle ("middle pin") in the protruding direction to perfuse the channel downstream only (or drive the middle pin in the retracting direction, if perfusing upstream). We used a CFW in the protruding direction only because the area of interest lies downstream (See **Figure S2A**). In this case, backflow can be suppressed by setting the discontinuity point of the CFW at the time when the downstream is completely blocked, as shown in **Figure S1B**.

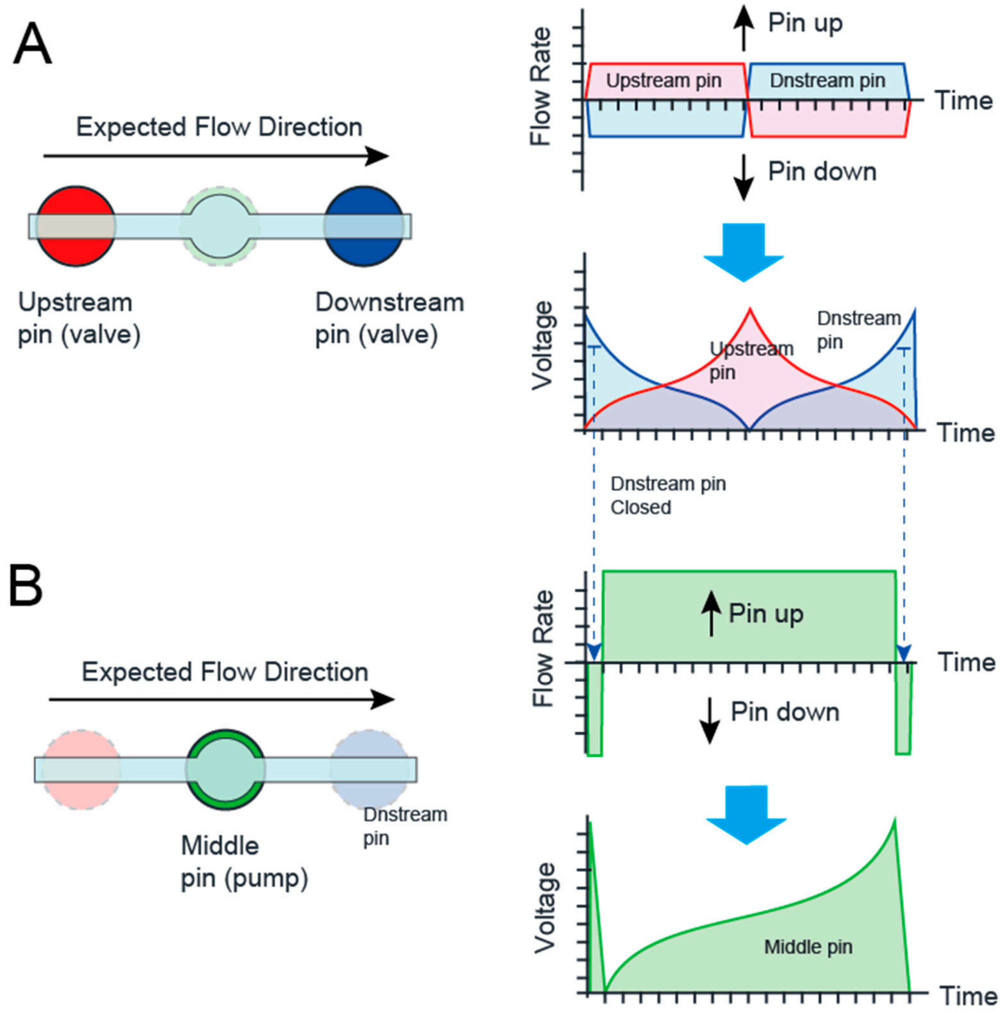

Figure S1. Construction of waveforms for a three-stranded Braille pin pump. Each pin's waveform is a repetition of the "constant flow rate" waveforms (CFWs) depicted in Fig. 2. The waveform for the pin at the middle contains a CFW in the upstroke direction only, whereas the upstream and downstream pins were driven with both upstroke and downstroke CFWs. The waveforms for the upstream and downstream pin are shifted half-cycle to each other.

SI. 2. A Y-shaped microchannel with integrated Braille-driven dual micropumps

We employed a simple microfluidic setup consisting of a bifurcated elastomeric microchannel with a Braille actuator, called a Braille cell, to analyze flowrates generated by Braille pin actuation. A Y-shaped microchannel feature fabricated by diffused light soft lithography (37) attached to a thin (300 μm-thick) PDMS membrane was sandwiched with a bottom coverglass and a 3D-printed fingerplate fixture, as shown in **Figure S2A**. Each branch of the Y-shaped channel has seats for three of the eight Braille cell pins. An actuated pin easily deforms the PDMS membrane to occlude the channel. **Figure S2B** depicts the mutual positional relationship of each component that constitutes the microchannel and the Braille cell.

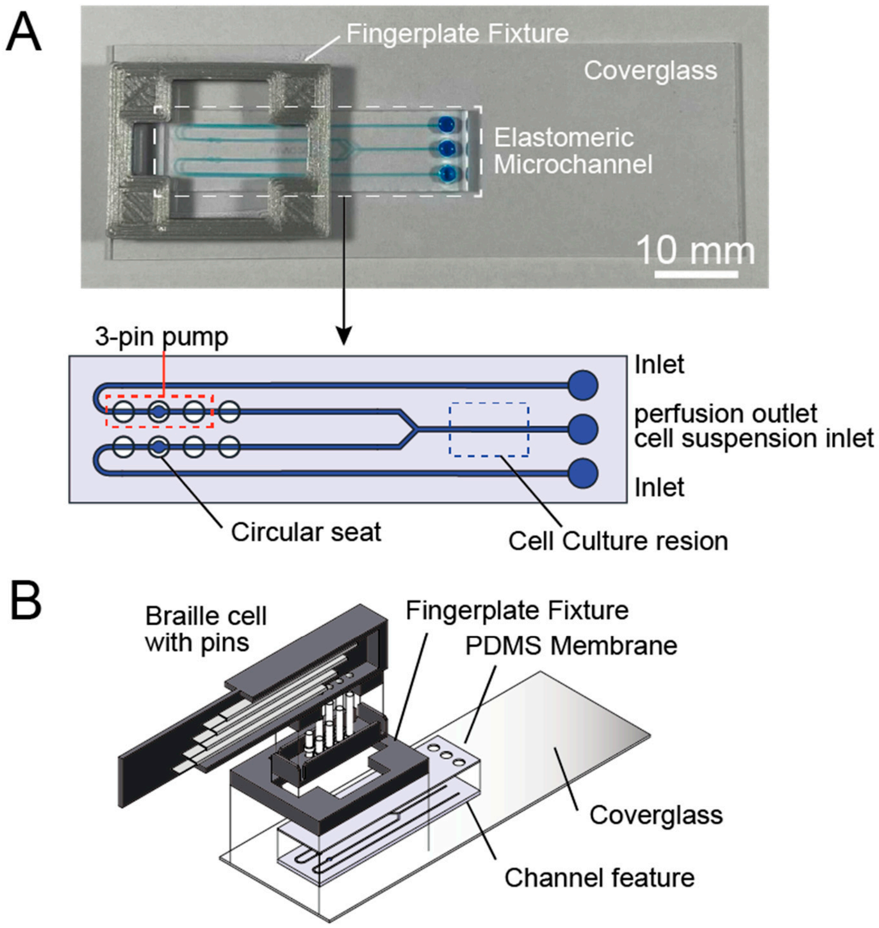

Figure S2. A Braille pump-driven microfluidic device used for flowrate evaluation. A) An upper view of the microfluidic device without a Braille actuator. B) Illustration of an exploded view of the microfluidic device with a Braille actuator attached.

SI. 3. MATLAB Script

The MATLAB script used to calculate the exclusion volume is shown below. The sliced video is imported into ImageJ, and the intensity in the sliced images was converted to the thickness of the fluorescent dye solution in the channel.

```
slices = 2500;

% m0: microchannel volume of the 1st frame
m0 = ComputeVolume('xxxx-0000.tif');

% Z: the volume of excluded fluid
Z = zeros(slices + 1, 1);

%Read fluorescence image
for i = 0:slices
    Imgfilename = sprintf('xxxx-%04d.tif', i);
    m = ComputeVolume(Imgfilename);
    Z(i+1,1) = m0 - m;
end

function m = ComputeVolume(fname)
    pixel = 1.66667;     %pixel length[um](5x)
    height = 50;          %microchannel width height length[um]

    I = imread(fname);

    j = im2double(I) * height;    %Converts fluorescence intensity to a range of 0~50um
    k = pixel^2 * j;              %Microchannel volume per pixel

    l = sum(k);
    m = sum(l);
end
```

SI. 4. Information of Fluid Structure Interaction Analysis

The fluid-structure interaction analysis in this paper was performed using the analysis systems Transient Structural Analysis, Fluent, and System Coupling in ANSYS Workbench. The typical settings for each analysis system are summarized in the table below.

Table S1. Analysis Settings for Transient Structural Analysis

<table>
<tr><td>Analysis System</td><td colspan="3">Transient Structural Analysis</td></tr>
<tr><td rowspan="2">Mesh</td><td colspan="2">type</td><td>tetrahedral</td></tr>
<tr><td colspan="2">size[mm]</td><td>0.1</td></tr>
<tr><td rowspan="3">Spring definition</td><td colspan="2">Scope</td><td>Top face of pin - Ground</td></tr>
<tr><td colspan="2">Longitudinal stiffness[N/mm]</td><td>0.252</td></tr>
<tr><td colspan="2">Pretension load</td><td>Free length</td></tr>
<tr><td rowspan="4">Contact definition</td><td rowspan="2">Pin - PDMS</td><td>type</td><td>No friction</td></tr>
<tr><td>Offset</td><td>0.01mm</td></tr>
<tr><td rowspan="2">Top of channel wall - Bottom of channel wall</td><td>type</td><td>No friction</td></tr>
<tr><td>Offset</td><td>0.005mm</td></tr>
<tr><td rowspan="4">Boundary condition</td><td colspan="2">Boundary surface between fluid and solid</td><td>Set over entire channel wall</td></tr>
<tr><td colspan="2">Fixed</td><td>Bottom surface of PDMS</td></tr>
<tr><td colspan="2" rowspan="2">Displacement</td><td>Top face of pin</td></tr>
<tr><td>Displacement waveform determined by table data.</td></tr>
<tr><td colspan="3">PDMS physical properties</td><td>SYLGARD184[38]</td></tr>
</table>

Table S2. Analysis Settings for Fluent

Analysis System	Fluent		
Mesh	type		Tetrahedral
	size[mm]		0.00125
Liquid Physical properties	Density[kg/m^3]		998.2(default)
	Viscosity[kg/(m・s)]		0.001003(default)
Boundary condition	WALL		Contact surface with PDMS
	OUTLET		Inlet and outlet of the channel.
			Gauge pressure 0[Pa]
Dynamic mesh	Mesh method		Smoothing and Remeshing
	Dynamic mesh zone	System coupling	Channel Wall
		Deforming	Channel Outlet
	Option	Contact detection	Top and bottom of channel
			Proximity threshold 0.015[mm]
		flow control	Create a new cell zone condition and set the viscous resistance to 1*10^12[m^{-2}].

Table S3. Analysis Settings for System coupling

Analysis System	System coupling
Step size[s]	0.01
Data transfer	Set between PDMS channel wall and fluid wall

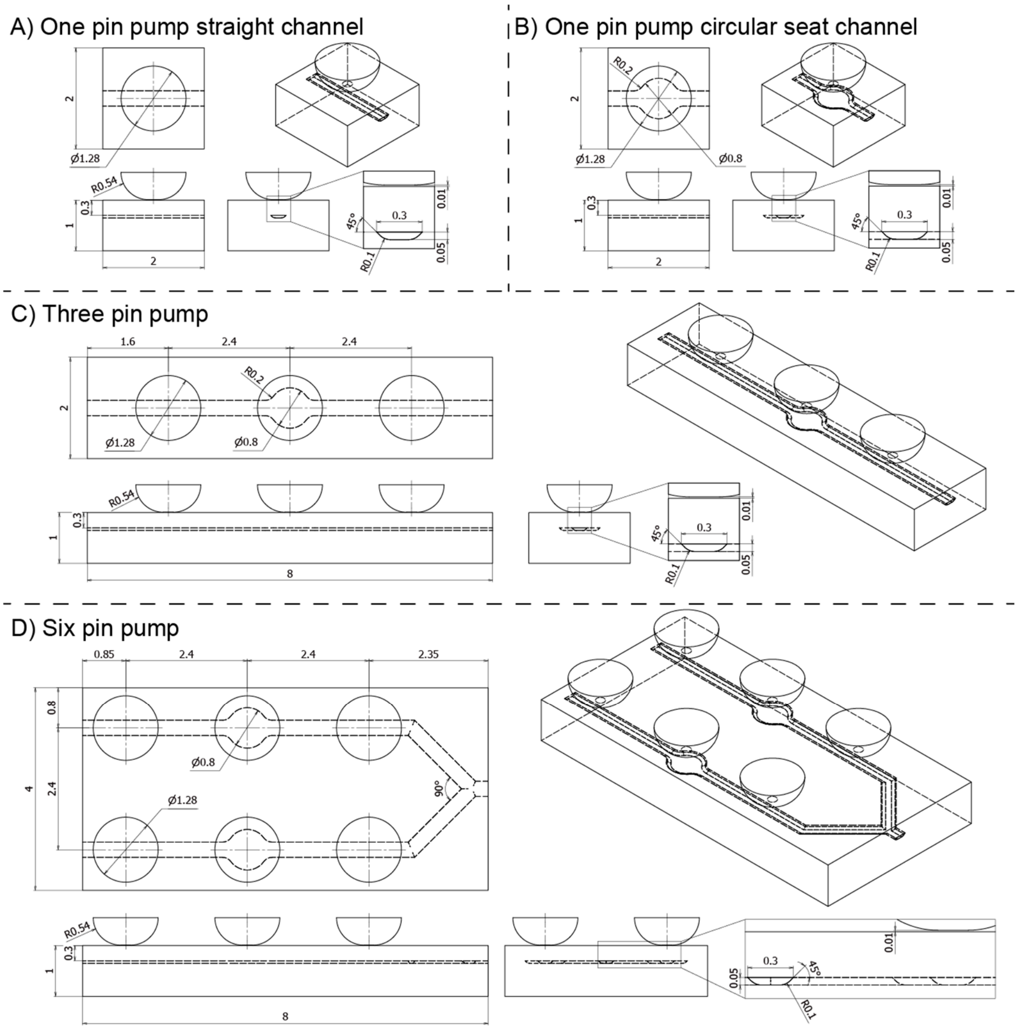

Figure S3. 3D model used for FSI analysis. A),B) Models used for the analysis of the 1-pin pump. C) Model used for the three-pin pump. D) Model used for the six-pin pump.

SI. 5. Configuration of 6-pin pump

As shown in Figure S4, the six-pin pump comprises two three-stranded Braille pin pumps in parallel and operated, and the same waveform drives the two pumps with half-cycle shifted to each other.

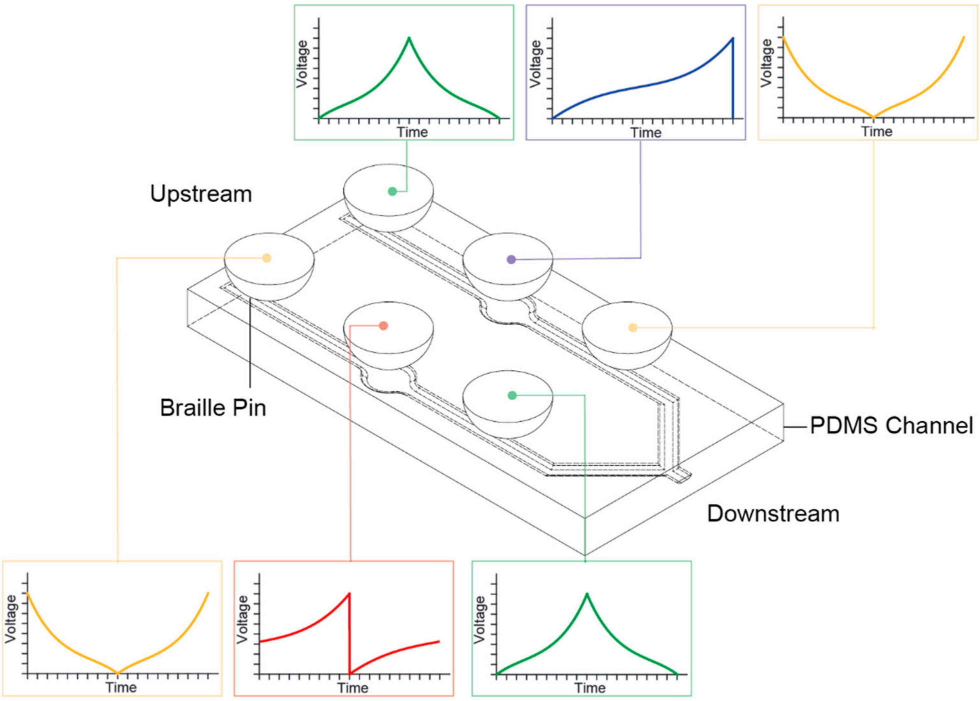

Figure S4. A parallel pair of two three-stranded Braille pin pump to eliminate backflow at the downstream. Two pins illustrated with waveforms of the same color denote that these pins were driven with an identical waveform.

SI. 6. Fabrication methods for microfluidic devices

**Standard Operation Procedures**				
**Project**		Braille micropump	**Date**	2022/01/30
**Deliverables**		Microfluidic device	**Revision**	1
**No.**	**Name**	**Description**	**Key points**	**Notes**
**Membrene**				
1.	Masking the glass	Clean the 100*100mm glass with ethanol and acetone, and cover the four sides with masking tape.	Apply the masking tape so that it protrudes from the glass.	Covering the four sides with masking tape prevents the PDMS from getting around the back of the glass after spin coating, making the subsequent process easier.
2.	Spin-coat	Set the glass in the spin coater. Drip PDMS prepolymer onto the glass and spin coat.	PDMS prepolymer is applied to the center of the glass in a circular pattern.	Rotation conditions for coating to 300μm<br>(350 rpm, 5.0 SLsec, 30.0 TMsec)
3.	Vacuum defoaming	Remove the glass from the spin coater and peel off the masking tape. Transfer the glass to a desiccator and degas for 30 minutes while pulling a vacuum.	Be careful of particles when venting the desiccator.	
4	Heat curing	Cured in an oven at 65°C for 3 hours, then cured in an oven at 120°C for 10 minutes.		
5	Mold release	Release the PDMS membrane from the glass.		
**Channel layer**				
1	Coat PDMS prepolymer	Coat PDMS prepolymer in a microchannel mold using a film applicator.		
2	Vacuum defoaming	Transfer the microchannel mold to a desiccator and degas for 30 minutes while pulling a vacuum.	Be careful of particles when venting the desiccator.	
3	Heat curing	Cured in an oven at 65°C for 3 hours, then cured in an oven at 120°C for 10 minutes.	Release the PDMS from the mold before transferring it to a 120°C oven.	
**Assembly**				

**Standard Operation Procedures**				
**Project**		Braille micropump	**Date**	2022/01/30
**Deliverables**		Microfluidic device	**Revision**	1
**No.**	**Name**	**Description**	**Key points**	**Notes**
1	Trim the channel layer and membrane layer	Trim the channel layer using a scalpel to match the dotted guide.<br>The membrane layer is trimmed larger than the channel layer.	Trim the film layer to a larger size to allow for misalignment during plasma bonding.	
2	Plasma Bonding	Both PDMS layers were bonded after exposure to vacuum air plasma at 200 Pa, 20 mA for 30 s.	Drill holes for inlets and outlets in the membrane layer using a φ1.5mm biopsy trepan in advance.	Temporarily fix the channel on the membrane layer and use a pen to mark the inlet outlet part of the channel to determine the drilling position.
3	Trim of the membrane layer	Trim the excess membrane using a scalpel to match the channel layer.		
4	Plasma Bonding	PDMS layer and coverglass were bonded after exposure to vacuum air plasma at 200 Pa, 20 mA for 30 s.	Use a pen to mark the location of the channel layer on the glass slide in advance.	
5	Bonding fingerplate fixture	Gluing the fingerplate fixture and coverglass together using epoxy adhesive.	Gluing is done while the finger plate is fixed to the fingerplate fixture.	The microchannel seen through the hole in the finger plate is observed under a microscope and aligned.
